# Prevalence of Clinical Obesity in US Adults Based on a Newly Proposed Definition

**DOI:** 10.1001/jamanetworkopen.2025.33806

**Published:** 2025-09-25

**Authors:** Dahyun Park, Dong Hoon Lee, Rockli Kim, Min-Jeong Shin, S. V. Subramanian

**Affiliations:** 1 Institute for BioMaterials, Korea University, Seoul, South Korea; 2 Department of Sport Industry Studies, Yonsei University, Seoul, South Korea; 3 Department of Nutrition, Harvard T.H. Chan School of Public Health, Boston, Massachusetts; 4 Division of Health Policy and Management, College of Health Science, Korea University, Seoul, South Korea; 5 Interdisciplinary Program in Precision Public Health, Department of Public Health Sciences, Graduate School of Korea University, Seoul, South Korea; 6 School of Biosystems and Biomedical Sciences, College of Health Science, Korea University, Seoul, South Korea; 7 Harvard Center for Population and Development Studies, Cambridge, Massachusetts; 8 Department of Social and Behavioral Sciences, Harvard T. H. Chan School of Public Health, Boston, Massachusetts

## Abstract

This cross-sectional study compares the prevalence of obesity among US adults using 2 definitions: a body mass index–based definition and a new evidence-based definition of clinical obesity focusing on direct measures of body fat and organ dysfunction and physiological impairment.

## Introduction

The Lancet Diabetes & Endocrinology Commission recently proposed a new evidence-based definition of clinical obesity incorporating direct measures of body fat and emphasizing the presence of organ dysfunction or physiological impairment due to excess adiposity.^1^ The present study compared the prevalence of body mass index (BMI)–based obesity and clinical obesity using the National Health and Nutrition Examination Survey (NHANES) to identify populations potentially misclassified by conventional BMI thresholds.

## Methods

This cross-sectional study was exempt from institutional review board approval and the requirement of informed consent and followed STROBE reporting guideline. We analyzed data from the 2017-2018 NHANES, the most recent nationally representative survey using multistage probability sampling design following National Center for Health Statistics guidelines. Adults (aged ≥20 years) who completed the physical examination component and had valid anthropometric measurements were included. BMI-based obesity was defined as BMI (calculated as weight in kilograms divided by height in meters squared) of 27.5 for non-Hispanic Asian participants and 30 or greater for all other adults. Following the Lancet Commission,^1^ clinical obesity was defined as meeting both anthropometric and clinical criteria (eTable and eFigure in Supplement 1). The analysis was conducted using Stata 18 (StataCorp) from February to July 2025, and a 2-sided *P* < .05 was considered significant. Results are presented as weighted percentages with 95% CIs. For more information, see the eMethods in Supplement 1.

## Results

A total of 4990 adults aged 20 years or older were included in the final analytic sample (weighted mean [SD] age, 48.12 [17.13] years; 51.69% [95% CI, 49.91%-53.47%] female; 15.43% [95% CI, 11.35%-19.51%] Hispanic; 11.38% [95% CI, 7.93%-14.83%] non-Hispanic Black; 62.71% [95% CI, 57.16%-68.26%] non-Hispanic White). The prevalence of BMI-based obesity was 43.81% (95% CI, 40.16%-47.47%), comparable to clinical obesity (44.74%; 95% CI, 41.73%-47.76%) (Table). Despite the similar prevalence estimated by the 2 definitions, only 25.76% (95% CI, 22.87%-28.66%) of the study population was classified as having both BMI-based and clinical obesity (Figure). The remaining 18.01% (95% CI, 15.77%-20.26%) were classified as having obesity only by BMI-based criteria, and 18.87% (95% CI, 16.98%-20.77%) only by clinical obesity criteria. The prevalence of clinical obesity increased with age, reaching 83.65% (95% CI, 78.25%-89.05%) among individuals aged 80 years or older. Clinical obesity was more prevalent among adults with lower-income (53.16%; 95% CI, 46.57%-59.75%) and lower-education (53.56%; 95% CI, 47.63%-59.49%), as well as among Asian (42.69%; 95% CI, 38.21%-47.17%) and Hispanic (46.10%; 95% CI, 41.75%-50.44%) adults. Among adults with excess adiposity, 45.67% (95% CI, 42.73%-48.61) were classified as having preclinical obesity, and this proportion declined with age, from 77.27% (95% CI, 73.08%-81.46%) among adults aged 20 to 29 years to 8.49% (95% CI, 5.19%-11.79%) at age 80 years or older. Among those with clinical obesity, both organ dysfunction and mobility limitations were higher in older adults, women, and socioeconomically disadvantaged groups.

**Table.** Prevalence of BMI–Based Obesity and Clinical Obesity

Characteristic	Obesity prevalence among NHANES participants, weighted % (95% CI)^a^				
	Independent classification^b^		Joint classification^c^^,^^d^		
	BMI-based obesity^e^	Clinical obesity^f^	BMI-based obesity only^e^	Clinical obesity only^f^	Both
Total	43.81 (40.16-47.47)	44.74 (41.73-47.76)	18.01 (15.77-20.26)	18.87 (16.98-20.77)	25.76 (22.87-28.66)
Sex					
Men	44.15 (38.93-49.38)	45.71 (42.00-49.42)	18.90 (15.95-21.85)	20.42 (17.14-23.71)	25.22 (21.55-28.88)
Women	43.49 (39.16-47.83)	43.84 (40.28-47.40)	17.18 (14.67-19.69)	17.43 (15.14-19.71)	26.27 (22.81-29.74)
Age group, y					
20-29	37.93 (29.35-46.51)	14.10 (10.77-17.44)	27.57 (20.49-34.66)	3.75 (1.88-5.62)	10.35 (7.48-13.23)
30-39	44.95 (40.05-49.85)	28.14 (22.69-33.59)	25.33 (19.65-31.01)	8.52 (5.87-11.16)	19.62 (15.36-23.88)
40-49	47.19 (41.74-52.64)	40.57 (36.94-44.21)	23.58 (18.95-28.21)	16.84 (13.53-20.16)	23.57 (19.37-27.76)
50-59	45.80 (40.85-50.75)	52.01 (44.87-59.14)	13.07 (8.88-17.26)	19.17 (16.20-22.13)	32.66 (26.58-38.74)
60-69	45.78 (38.30-53.26)	65.89 (59.36-72.42)	10.38 (6.58-14.18)	30.52 (24.68-36.36)	35.37 (27.71-43.03)
70-79	45.43 (37.87-52.99)	78.81 (73.68-83.94)	5.00 (2.08-7.91)	38.19 (31.03-45.36)	40.30 (33.82-46.79)
≥80	31.41 (25.78-37.03)	83.65 (78.25-89.05)	0.85 (0.00-2.17)	52.79 (45.33-60.25)	30.42 (24.53-36.31)
Race and ethnicity					
Hispanic	42.99 (37.60-48.37)	46.10 (41.75-50.44)	16.70 (13.36-20.03)	19.77 (16.41-23.12)	26.28 (22.50-30.06)
Non-Hispanic Asian	49.82 (47.05-52.58)	42.69 (38.21-47.17)	20.47 (17.63-23.31)	13.34 (10.14-16.53)	29.35 (26.38-32.32)
Non-Hispanic Black	37.68 (33.26-42.11)	39.47 (33.36-45.58)	16.14 (12.90-19.38)	17.68 (13.21-22.15)	21.48 (15.77-27.19)
Non-Hispanic White	52.20 (47.73-56.67)	40.25 (36.52-43.98)	26.59 (22.52-30.67)	14.46 (11.36-17.57)	25.50 (22.16-28.85)
Other^g^	32.97 (29.03-36.92)	41.46 (36.74-46.18)	14.75 (12.55-16.96)	23.29 (18.45-28.12)	18.17 (15.10-21.24)
Income quartile					
1 (Lowest)	42.92 (36.91-48.93)	53.16 (46.57-59.75)	13.01 (10.09-15.93)	23.20 (18.73-27.68)	29.88 (25.26-34.51)
2	45.92 (39.30-52.54)	51.28 (45.64-56.92)	15.48 (12.08-18.88)	20.80 (15.98-25.63)	30.39 (25.62-35.16)
3	46.24 (39.30-53.17)	43.32 (36.71-49.93)	19.66 (15.03-24.28)	16.62 (14.10-19.14)	26.50 (20.30-32.71)
4 (Highest)	41.88 (36.49-47.27)	39.76 (35.70-43.82)	19.81 (15.66-23.96)	17.68 (14.39-20.97)	22.06 (18.79-25.33)
Education					
<High school	41.96 (37.16-46.76)	53.56 (47.63-59.49)	14.10 (10.88-17.32)	25.52 (21.41-29.63)	27.74 (23.42-32.06)
High school graduate	48.27 (44.26-52.28)	47.54 (41.20-53.88)	18.53 (14.55-22.51)	17.67 (13.90-21.44)	29.67 (26.07-33.26)
Some college	48.70 (44.61-52.79)	47.16 (42.29-52.02)	19.63 (16.61-22.65)	18.10 (15.77-20.42)	29.06 (24.02-34.10)
≥College graduate	35.72 (29.85-41.59)	36.72 (33.64-39.79)	17.35 (13.53-21.17)	18.29 (15.73-20.86)	18.37 (15.01-21.73)

Abbreviations: BMI, body mass index; NHANES, National Health and Nutrition Examination Survey.

^a^ Prevalence estimates are weighted and presented as percentages with 95% CIs, accounting for the complex survey design of NHANES.

^b^ Independent classification refers to the overall prevalence of BMI-based obesity and clinical obesity separately, regardless of overlap.

^c^ Joint classification identifies the proportion of individuals classified exclusively by BMI, exclusively by clinical criteria, or by both definitions simultaneously.

^d^ All group-wise differences in obesity prevalence under the joint classification were statistically significant based on the Wald test (*P* < .001).

^e^ BMI-based obesity was defined as a BMI of 27.5 or greater for non-Hispanic Asian participants and 30 or greater for all other adults. BMI was calculated as weight in kilograms divided by height in meters squared.

^f^ Clinical obesity was defined based on the presence of excess adiposity along with signs or symptoms of organ dysfunction or limitations in daily functioning, using operational indicators from NHANES (see the eTable in Supplement 1). Signs and symptoms included self-reported or measured limitations related to cardiovascular, metabolic, hepatic, kidney, or neurologic conditions, or difficulty walking, standing, or performing related activities.

^g^ Other included any race or ethnicity not otherwise specified.

**Figure.**
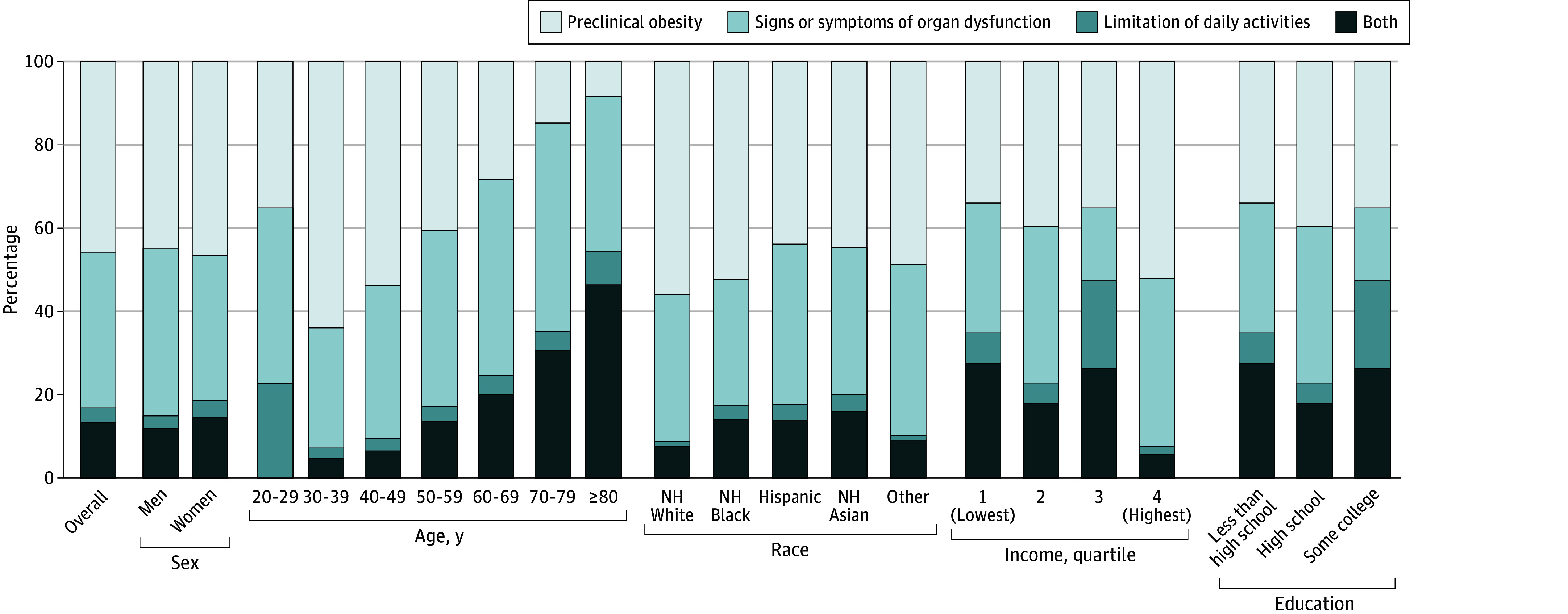
Proportion of Clinical Obesity and Preclinical Obesity Among Participants With Excess Adiposity, Stratified by Demographic and Socioeconomic Characteristics Prevalence estimates reflect the weighted proportion of the US noninstitutionalized adult population. Clinical obesity was defined based on the presence of excess adiposity and evidence of organ dysfunction or limitations in daily activities, using operationalized indicators from the National Health and Nutrition Examination Survey (see the eTable in Supplement 1). The “organ dysfunction group represents clinical obesity with signs or symptoms of organ dysfunction only; the daily activity limitation group represents clinical obesity with limitations in daily activities only; and the both group represents clinical obesity with both organ dysfunction and daily activity limitations. NH indicates non-Hispanic.

## Discussion

This cross-sectional study comparing obesity prevalence defined by BMI-based criteria and clinical obesity framework has several important implications. First, although the prevalence of clinical obesity was similar to that of BMI-based classification, the 2 definitions identified distinct populations, indicating notable discordance. The clinical obesity framework suggests that timely intervention including pharmacotherapy, bariatric surgery, or lifestyle modifications may reduce obesity-related burden more effectively than what BMI-based estimates suggest.

Second, younger adults and those with higher socioeconomic status were more often classified as having BMI-based obesity only, consistent with prior evidence that BMI and other adiposity measures yield similar prevalence estimates in this age group.^2^ In contrast, older adults were more frequently classified as having clinical obesity despite lower BMI, reflecting the greater burden of comorbidities and functional decline not captured by BMI alone.

Finally, approximately 45.67% of adults with excess adiposity did not exhibit evident organ dysfunction or substantial physical limitations, and were therefore classified as having preclinical obesity. Given their elevated risk of progressing to clinical obesity and associated chronic disease conditions, early-stage preventive interventions are crucial for effective obesity management.^3^

Collectively, our findings provide valuable insights into identifying individuals with obesity using different classifications. Limitations include that NHANES measures may not fully capture all aspects of clinical obesity as defined by the Lancet Commission. Although this study focused on adults, future research should explore clinical obesity in pediatric population to more comprehensively address the obesity epidemic and its burden from excess adiposity though more precise prioritization of public health policies and therapeutic approaches.

## Notes

Supplement 1. **eTable.** Criteria for Clinical Obesity Based on the Lancet Commission Definition and Their Operationalization Using Variables From the National Health and Nutrition Examination Survey **eFigure.** Flowchart of Diagnosing Pre-Clinical and Clinical Obesity
eMethods.

Supplement 2.
Data Sharing Statement
