# Supplementary material for: Prevalence of Clinical Obesity in US Adults Based on a Newly Proposed Definition
Source: JAMA Netw Open. 2025 Sep 25;8(9):e2533806. doi: 10.1001/jamanetworkopen.2025.33806 (PMC12464785; doi:10.1001/jamanetworkopen.2025.33806)
Supplement: Supplement 1. — eTable. Criteria for Clinical Obesity Based on the Lancet Commission Definition and Their Operationalization Using Variables From the National Health and Nutrition Examination Survey eFigure. Flowchart of Diagnosing Pre-Clinical and Clinical Obesity eMethods. [file jamanetwopen-e2533806-s001.pdf]

Supplemental Online Content

Park D, Lee DH, Kim R, Shin MJ, Subramanian SV. Prevalence of clinical obesity in US adults based on a newly proposed definition. *JAMA Netw. Open*. 2025;8(9):e2533806. doi:10.1001/jamanetworkopen.2025.33806

eTable. Criteria for Clinical Obesity Based on the Lancet Commission Definition and Their Operationalization Using Variables From the National Health and Nutrition Examination Survey

eFigure. Flowchart of Diagnosing Pre-Clinical and Clinical Obesity

eMethods. Flow of information through the different phases of the review

This supplemental material has been provided by the authors to give readers additional information about their work.

© 2025 Park D et al. *JAMA Network Open*.

eTable. Criteria for clinical obesity based on the Lancet Commission Definition and their operationalization using variables from the National Health and Nutrition Examination Survey

Step 1. Excess adiposity

Excess adiposity was defined as meeting any of the following four criteria

1	BMI plus at least one additional anthropometric measure (waist circumference, waist to hip ratio, or waist to height ratio)
2	Two anthropometric measures in combination regardless of BMI
3	Total body fat by direct body fat measurement, such as a dual-energy X-ray absorptiometry scan
4	BMI ≥ 40 kg/m^2

Criteria by measurement	Male	Female
BMI	$\geq$ 30 kg/m^2(Asian: $\geq$ 27.5 kg/m^2)	$\geq$ 30 kg/m^2(Asian: $\geq$ 27.5 kg/m^2)
Waist circumference	$\geq$ 102cm (Asian: $\geq$ 90 cm)	$\geq$ 88cm (Asian: $\geq$ 80 cm)
Waist-to-hip ratio	> 0.9	> 0.85
Waist-to-height ratio	> 0.5	> 0.5
Total body fat	> 25%	> 35%

Step 2. Clinical obesity

Clinical obesity was defined as the presence of organ system dysfunction or impairments in mobility and activities of daily living

	Organ, tissue, or body system	Diagnostic criterion (as agreed by commissioners)	NHANES based variables
1	Central nervous system	Signs of raised intracranial pressure such as vision loss and/or recurrent headaches	Visual impairment (self-reported)
2	Upper airways	Apnoeas/hypopnoeas during sleep due to increased upper airways resistance	Sleep apnea (self-reported)
3	Respiratory	Hypoventilation and/or breathlessness and/or wheezing due to reduced lung and/or diaphragmatic compliance	Not assessed
4	Cardiovascular (ventricular)	Reduced left ventricular systolic function - Heart failure with reduced ejection fraction	Congestive heart failure (self-reported)
5	Cardiovascular (atrial)	Chronic/recurrent atrial fibrillation	Not assessed
6	Cardiovascular (pulmonary)	Pulmonary artery hypertension	Not assessed
7	Cardiovascular	Chronic fatigue, lower limb edema due to impaired diastolic dysfunction: Heart failure with preserved ejection fraction	Not assessed
8	Cardiovascular (thrombosis)	Recurrent deep vein thrombosis and/or pulmonary thromboembolic disease	Not assessed
9	Cardiovascular (arterial)	Raised arterial blood pressure	Blood pressure (measured), Hypertension medication (self-reported)
10	Metabolism	The cluster of hyperglycemias, high TG levels, and low HDL-C levels	FPG, TG, HDL-C (measured), Glucose or cholesterol medication (self-reported)
11	Liver	NAFLD with hepatic fibrosis	NAFLD Fibrosis Score (measured), Liver fibrosis (self-reported)
12	Renal	Microalbuminuria with reduced eGFR	eGFR (measured)
13	Urinary	Recurrent/chronic urinary incontinence	Urinary incontinence frequency (self-reported)
14	Reproductive (female)	Anovulation, oligo-menorrhea and polycystic ovary syndrome	Not assessed
15	Reproductive (male)	Male hypogonadism	Not assessed
16	Musculoskeletal	Chronic, severe knee or hip pain associated with joint stiffness and reduced range of joint motion	Difficulty with stooping, crouching, or kneeling (self-reported)
17	Lymphatic	Lower limbs lymphedema causing chronic pain and/or reduced range of motion	Not assessed
18	Limitations of daily activities	Significant, age-adjusted limitations of mobility and/or other basic activities of daily living	Difficulty with walking, standing, sitting, or daily activities (self-reported)

Note: BMI, Body mass index; NHANES, National health and nutrition examination survey; FPG, Fasting plasma glucose; TG, Triglycerides; HDL-C, High-density lipoprotein cholesterol; NAFLD, Nonalcoholic fatty liver disease; eGFR, Estimated glomerular filtration rate.

© 2025 Park D et al. *JAMA Network Open*.

eFigure. Flowchart of diagnosing pre-clinical and clinical obesity

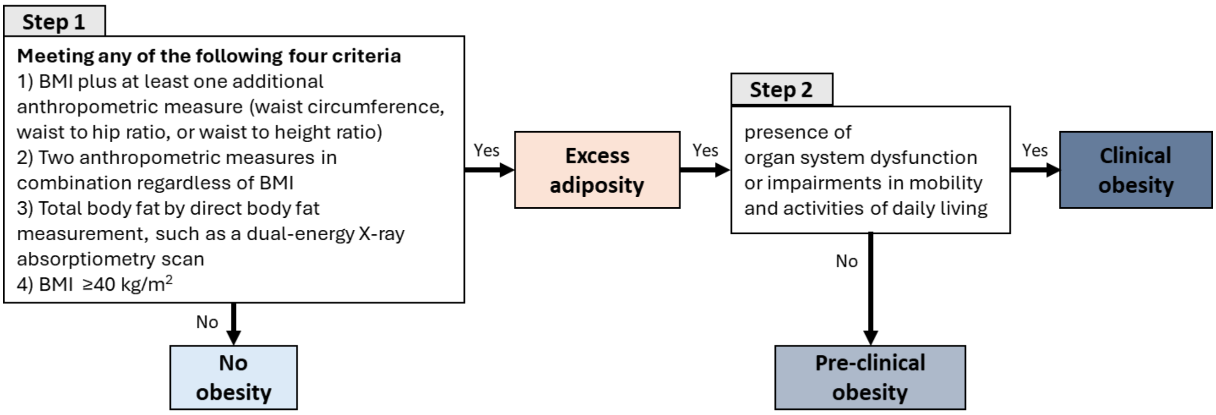

© 2025 Park D et al. *JAMA Network Open*.

eMethods

Participants with missing or implausible anthropometric data were excluded. Excluded individuals did not differ meaningfully from those included in terms of key demographic characteristics, including sex, race/ethnicity, and income level. Race and ethnicity data were collected as part of the (National Health and Nutrition Examination Survey) NHANES survey. Race and ethnicity categories included Hispanic, non-Hispanic Asian, non-Hispanic Black, non-Hispanic White, and other race or ethnicity [defined as any race or ethnicity not otherwise specified]). Race and ethnicity were included to ensure nationally representative estimates consistent with NHANES reporting standards. BMI-based obesity was defined as BMI (calculated as weight in kilograms divided by height in meters squared) of 27.5 for non-Hispanic Asian participants and 30 or greater for all other adults, applied consistent with WHO recommendations and the standard adopted in the Lancet Commission definition, which reflect higher metabolic risk at lower BMI levels in Asian populations. Clinical obesity was defined based on the *Lancet* Commission criteria, which require the presence of both anthropometric excess adiposity and clinical indicators of organ system dysfunction or physical impairment attributable to excess adiposity (eTable and eFigure). All analyses were conducted using Stata version 18 (StataCorp), incorporating sampling weights, strata, and primary sampling units to account for the complex survey design and ensure nationally representative estimates. Categorical variables were compared using design-adjusted Wald tests or Rao–Scott chi-square tests, and continuous variables were evaluated using survey-weighted linear regression models. All statistical tests were two-sided, and $P < .05$ was considered statistically significant. This study used publicly available, de-identified data and was therefore exempt from institutional review board (IRB) approval.

© 2025 Park D et al. *JAMA Network Open*.
