# Supplementary material for: Prevalence of Clinical Obesity in US Adults Based on a Newly Proposed Definition
Source: JAMA Netw Open. 2025 Sep 25;8(9):e2533806. doi: 10.1001/jamanetworkopen.2025.33806 (PMC12464785; doi:10.1001/jamanetworkopen.2025.33806)
Supplement: Supplement 2. — Data Sharing Statement [file jamanetwopen-e2533806-s002.pdf]

Data Sharing Statement

Park. Prevalence of Clinical Obesity in US Adults Based on the Newly Proposed Definition. *JAMA Netw Open*. Published September 25, 2025. doi:10.1001/jamanetworkopen.2025.33806

Data
Data available: No

Additional Information
Explanation for why data not available: The National Health and Nutrition Examination Survey data are publicly available from the CDC website: https://wwwn.cdc.gov/nchs/nhanes/
